# Optical coherence tomography and imaging biomarkers as outcome predictors in diabetic macular edema treated with dexamethasone implant

**DOI:** 10.1038/s41598-022-07604-7

**Published:** 2022-03-09

**Authors:** Hung-Da Chou, Cheng-Hsiu Wu, Wei-Yu Chiang, Nan-Ni Chen, Yih-Shiou Hwang, Kuan-Jen Chen, Chien-Hsiung Lai, Pei-Chang Wu, Yi-Hao Chen, Ling Yeung, Shih-Chieh Shao, Chi-Chun Lai, Wei-Chi Wu

**Affiliations:** 1 Department of Ophthalmology, Chang Gung Memorial Hospital, Linkou Medical Center, No. 5, Fuxing St., Gueishan Dist., Taoyuan City, 333 Taiwan; 2 College of Medicine, Chang Gung University, Taoyuan City, Taiwan; 3 Department of Ophthalmology, Keelung Chang Gung Memorial Hospital, Keelung City, Taiwan; 4 Department of Ophthalmology, Kaohsiung Chang Gung Memorial Hospital, Kaohsiung City, Taiwan; 5 Department of Ophthalmology, Chiayi Chang Gung Memorial Hospital, Chiayi City, Taiwan; 6 Department of Ophthalmology, Chang Gung Memorial Hospital, Xiamen Branch, Xiamen, China; 7 Department of Ophthalmology, Jen-Ai Hospital Dali Branch, Taichung, Taiwan; 8 Department of Nursing, Chang Gung University of Science and Technology, Chiayi, Taiwan; 9 School of Traditional Chinese Medicine, College of Medicine, Chang Gung University, Taoyuan City, Taiwan; 10 Department of Pharmacy, Keelung Chang Gung Memorial Hospital, Keelung City, Taiwan

**Keywords:** Biological techniques, Biomarkers

## Abstract

In this retrospective, multicenter study, we determined the predictive value of imaging biomarkers in diabetic macular edema (DME) outcomes following dexamethasone (DEX) implant(s). Sixty-seven eyes of 47 patients’ best-corrected visual acuity (BCVA) and central foveal thickness (CFT) on optical coherence tomography (OCT) before and after intravitreal DEX implants were evaluated. Baseline imaging biomarkers were graded using fundus photography and OCT, and the predictive value of biomarkers for significant treatment effects at six months was analyzed. Six months after 2.0 ± 0.8 (mean ± SD) DEX implants, 35 (52%) and 16 (24%) eyes had CFT reduction ≥ 10% from baseline and decreased to < 300 µm, respectively. BCVA improved ≥ 3 lines in 15 (22%) and remained stable in 38 (57%) eyes. At six months, eyes with severe intraretinal cyst (IRC), abundant hyperreflective dots (HRD), and moderate or severe hard exudate had a significantly higher chance of CFT reduction ≥ 10%. Eyes with abundant HRD at baseline and those underwent three DEX implants were more likely to achieve CFT < 300 µm. Eyes with DME and severe IRC, abundant HRD, or moderate-to-severe hard exudate at baseline were more likely to show a significant reduction in CFT six months after DEX implant.

## Introduction

Diabetes is a metabolic disease that affects over 422 million people worldwide^1^. Among the main complications of diabetes, diabetic retinopathy and diabetic macular edema (DME) are the leading causes of visual impairment in working-age people.

Anti-vascular endothelial growth factor (anti-VEGF) drugs have been widely used as a treatment for DME and improved visual outcomes. However, some patients do not respond to anti-VEGF. A post hoc analysis showed that nearly 40% of the eyes resulted in suboptimal best-corrected visual acuity (BCVA) improvement of less than five letters after three months of anti-VEGF treatment^2^. In addition, a study showed that after six months of a monthly injection, 32–66% of the treated eyes did not respond to anti-VEGF, resulting in persisting edema and reduced visual acuity (VA)^3^. Since inflammation is tightly involved in the development and worsening of DME^4^, the use of steroids has emerged as an alternative treatment^5^.

Intravitreal dexamethasone (DEX) implants (Ozurdex, Allergan, Irvine, CA) have been applied since 2009 to treat different ocular diseases. After the injection, the implant slowly dissolves in the vitreous cavity and provides a sustained release of DEX for up to six months. It is indicated for macular edema associated with retinal vein occlusions, noninfectious uveitis^6^, macular edema associated with diabetes^7,8^, and diabetic tractional retinal detachment^9^.

Studies have shown that DEX implants are effective in naive eyes as well as those that responded poorly to anti-VEGF^10^ and that several non-invasive imaging characteristics, or so-called imaging biomarkers, could predict these outcomes^11–14^. Although some of the earlier studies were either limited by patient number^15–17^, or short-term follow-up^14,18^, later real-life multicenter studies with bigger samples have shown the importance of the use of imaging biomarkers to ascertain outcomes in DME patients following DEX implants^19^. Our study aimed to evaluate the predictive value of imaging biomarkers for outcomes in Asian DME patients following DEX implants in a real-world, multicenter scenario.

## Materials and methods

A retrospective multicenter, single-arm study was conducted between June 2017 and February 2020 in four branch hospital sites in Taiwan. The inclusion criteria were patients with diabetes type 1 or 2 who presented a vision decline and DME and were treated with intravitreal DEX implant(s). DME was diagnosed based on a central foveal thickness (CFT) over 300 µm by spectral-domain optical coherence tomography (SD-OCT; Spectralis; Heidelberg Engineering, Heidelberg, Germany) measured with the built-in software. The Institutional Review Board approved the study (No. 201600962A3C101 from the Chang Gung Memorial Hospital) and adhered to the tenets of the Declaration of Helsinki. The Chang Gung Memorial Hospital Institutional Review Board also waived the requirement for informed consent due to the retrospective nature of this study.

The exclusion criteria were patients who received anti-VEGF for less than one month or macular grid laser/intravitreal corticosteroids for less than three months before the baseline (i.e., the time a patient received the first DEX implant), had concurrent macular pathologies or prior intraocular surgeries except for cataract extraction, or were followed for less than six months. Patients who received treatments for DME other than DEX implant and vitrectomies during the six-month follow-up period were also excluded.

Demographics, diabetic status, and systemic and ocular conditions were obtained from medical records. HbA1c levels were documented if measured less than three months before the baseline. Ophthalmic examinations, including BCVA, intraocular pressure (IOP), slit-lamp biomicroscopy, color fundus photographs, and SD-OCT images, were collected at baseline and six months after the first DEX implant.

### DME and imaging biomarkers grading

Four unmasked graders (HDC, CHW, WYC, NNC) graded the imaging biomarkers. For each biomarker, two graders were needed to reach a consensus, and a third one to arbitrate in case of discrepancy between the first two graders. Color fundus photographs were used to determine whether the eye underwent pan-retinal photocoagulation (PRP) and grade hard exudate (HE) status according to the Early Treatment Diabetic Retinopathy Study (ETDRS)^20^. Tomographic qualitative and quantitative OCT parameters were graded according to a recently described DME grading protocol^21^, which includes CFT, intraretinal cysts (IRC), subretinal fluid (SRF), the ellipsoid zone (EZ) status, disorganization of the inner retinal layers (DRIL), hyperreflective dots (HRD), and vitreoretinal relationship. Early, advanced, severe, and atrophic were the four distinct stages of DME, based on the four previously mentioned parameters (CFT, IRC, EZ, and DRIL).

### Study outcomes and endpoints

Snellen BCVA measurements were converted to logarithm of the minimum angle of resolution for statistical analysis. The main outcomes were evaluated six months after the first DEX implant. The three endpoints of the study were: (1) decrease in CFT greater than 10% from baseline; (2) decrease in CFT to less than 300 µm; and (3) improvement in BCVA of three or more ETDRS lines.

### Statistical analysis

Categorical and continuous variables is presented as No. (%) and mean ± standard deviation, respectively. The interrater reliability (interclass correlation coefficient) of the biomarker grading among the initial 2 graders were calculated by using the two-way mixed effects model for all pooled biomarkers gradings. The pre-and post-treatment conditions were compared by the Wilcoxon signed-rank test. A univariate logistic regression analysis was performed under the generalized estimating equations (GEE) framework to assess factors related to outcomes, considering that both eyes from an individual could be enrolled. For the GEE models with multivariate adjustments, age, sex, HbA1c level, and predictors with a *P* < 0.1 from the univariate models were included. All the analyses were performed using SAS Enterprise Guide Version 7.1. Statistical significance was considered with *P* < 0.05 for two-tailed tests.

## Results

Demographic and baseline characteristics of the study population are shown in Table 1. The study included 67 eyes of 47 patients, with a mean age of 67 and a mean HbA1c of 7.6%. Half of the eyes underwent prior PRP, and 11 eyes (16%) received anti-VEGF intravitreal injections more than one month before the DEX implant. The rest of the eyes (56 eyes, 84%) were all anti-VEGF naïve. The mean time since last anti-VEGF injection was 954 days (range, 42–3137 days) and the mean duration of DME was 109 days. Five eyes (8%) had glaucoma and were receiving IOP-lowering medication at the study baseline.

**Table 1:** Demographic and baseline characteristics.

Characteristics	Baseline
No. of patients	47
No. of eyes	67
Age, mean ± SD, y	66.6 ± 8.2
Male, No. (%)	29 (62)
Under renal dialysis, No. (%)	1 (2)
Hypertension, No. (%)	19 (40)
Prior myocardial infarction, No. (%)	6 (13)
Prior stroke, No. (%)	2 (4)
**Diabetis mellitus duration, No. (%)**	
5 y or less	8 (12)
5–15 y	19 (29)
More than 15 y	6 (9)
Not recorded	34 (51)
HbA1c (%), mean ± SD	7.6 ± 1.3
**Diabetis retinopathy grading, No. (%)**	
Mild or moderate NPDR	5 (8)
Severe or very severe NPDR	17 (25)
PDR	11 (16)
High-risk PDR	9 (13)
Cannot grade^a^	25 (37)
Diabetis retinopathy duration^b^, mean ± SD, y	5.1 ± 6.2
Diabetic macular edema duration, mean ± SD, d	109 ± 134
Prior PRP, No. (%)	33 (50)
Intravitreal anti-VEGF treated, No. (%)	11 (16)
Last intravitreal anti-VEGF injection time before DEX implant, mean ± SD, d	954 (1124)
Glaucoma status, No. (%)	5(8)

^a^Eyes with prior PRP were non-gradable except for those with neovascularization.

^b^Valid *n* = 48.

Abbreviation: *anti-VEGF* anti-vascular endothelial growth factor, *DEX implant* dexamethasone intravitreal implant, *NPDR* nonproliferative diabetic retinopathy, *PDR* proliferative diabetic retinopathy, *PRP* panretinal photocoagulation.

### Imaging biomarker grades at study baseline

Details of the baseline imaging biomarkers are listed in Table 2. The interrater reliability for biomarker gradings was moderate (interclass correlation coefficient: 0.56, 95% CI, 0.44–0.66). Forty-one eyes (62%) had CFT over 390 µm, 20 (30%) had severe IRC, and 14 (21%) had SRF in the fovea. The majority (63%) of the eyes had an intact EZ; however, DRIL was present in 74%. HRD was graded as abundant (higher than 30 dots) in nearly half of the eyes (45%). Half of the eyes (49%) had an epiretinal membrane. Combining the CFT, IRC, EZ, and DRIL status, the resulting DME staging was: early (17%), advanced (57%), and severe (12%) DME. Fundus photographic grading showed that 22 eyes (33%) had moderate or severe HE at baseline.

**Table 2:** Baseline DME imaging biomarkers.

DME imaging biomarkers	Baseline
**Central foveal thickness, No. (%)**	
300–329 µm	10 (15)
330–389 µm	16 (24)
≥ 390 µm	41 (62)
**Intraretinal cysts, No. (%)**	
Absent	6 (9)
Mild	15 (23)
Moderate	26 (39)
Severe	20 (30)
Presence of subretinal fluid, No. (%)	14 (21)
**Ellipsoid zone status, No. (%)**	
Intact	42 (63)
Disrupted	15 (23)
Absent	8 (12)
Cannot grade	2 (3)
Presence of DRIL, No. (%)	49 (74)
**Hyperreflective dots, No. (%)**	
Absent or scarce (< 30)	37 (56)
Abundant (> 30)	30 (45)
**Vitreoretinal relationship**	
Attached vitreous cortex in the fovea	19 (28)
Detached vitreous cortex in the fovea	10 (15)
Epiretinal membrane	33 (49)
Cannot grade	5 (8)
**Hard exudates, No. (%)**	
Absent	30 (45)
Mild	12 (18)
Moderate	12 (18)
Severe	10 (15)
Cannot grade	2 (3)
**DME stage**^**a**^**, No. (%)**	
Early DME	11 (17)
Advanced DME	38 (57)
Severe DME	8 (12)
Atrophic maculopathy	8 (12)
Cannot grade	2 (3)

^a^DME stage was graded according to Panozzo et al.’s^21^ study.

Abbreviation: *DME* diabetic macular edema, *DRIL* disorganized retinal inner layers.

### Anatomical and functional outcomes at six months

At six months, the eyes received an average of 2.0 ± 0.8 DEX implants (Table 3). Anatomically, CFT showed a significant reduction from pre-operative 459.9 ± 146.3 µm to post-operative 360.5 ± 127.4 µm (*P* < 0.001), and 35 eyes (52%) surmount the endpoint of CFT reduction ≥ 10% from baseline, with 16 eyes (24%) achieving CFT ≤ 300 µm after six months. Although the functional outcome showed no overall significant improvement in BCVA (*P* = 0.35), 15 eyes (22%) improved three lines or more, and 38 (57%) remained stable vision. Four eyes (6%) showed cataract progression over two grades after six months, but none underwent cataract surgery. The mean IOP through the study period significantly increased from 14 to 17 mmHg (*P* = 0.003); nevertheless, only five eyes (9%) exceeded 25 mmHg. Notably, two eyes (3%) had persistently elevated IOP despite maximal medical treatment and underwent transscleral cyclophotocoagulation.

**Table 3:** Six-month outcomes in eyes with diabetic macular edema treated by DEX implants.

	Values	*p-*value^a^
No. of eyes	67	–
DEX implant number mean ± SD	2.0 ± 0.8	–
CFT, mean ± SD, µm		< 0.001
Baseline	459.9 ± 146.3	
6-mo^a^	360.5 ± 127.4	
CFT change at 6-mo^b^		–
Decreased 10% or more from baseline	35 (52)	
Decreased to < 300 µm	16 (24)	
BCVA, mean ± SD, logMAR		0.35
Baseline	0.80 ± 0.37	
6-mo^c^	0.79 ± 0.49	
BCVA change at 6-mo		–
Improved 15 ETDRS letters or more	15 (22)	
Decreased 15 ETDRS letters or more	8 (12)	
Stable	38 (57)	
Missing	6 (9)	
Lens status, phakic, No. (%)		1.0
Baseline	22 (33)	
6-mo^c^	22 (33)	
Cataract progression	4 (6)	
IOP, mean ± SD, mmHg		0.003
Baseline	14.2 ± 3.7	
6-mo^c^	17.7 ± 5.1	
Elevated IOP^b^, No. (%)		–
> 25 mmHg	5 (9)	
> 35 mmHg	2 (3)	
Uncontrolled glaucoma needing intervention, No. (%)	2 (3)^d^	–

^a^Between the baseline and the 6-mo values.

^b^Valid *n* = 58.

^c^Valid *n* = 61.

^d^Two eyes from the same patient with persistent elevated IOP and needed argon laser trabeculoplasty.

Abbreviation: *BCVA* best-corrected visual acuity, *CFT* central foveal thickness, *DEX implant* dexamethasone intravitreal implant, *IOP* intraocular pressure, *logMAR* logarithm of the minimum angle of resolution.

### Univariate analysis of the biomarkers related to outcomes after DEX implants

To simplify the analysis, the grades of certain biomarkers, including IRC, EZ, and HE, were combined. Then, univariate logistic analysis of biomarkers was performed to select those that predict the outcomes after DEX implants (Table 4). A reduction in CFT ≥ 10% from baseline values could be predicted by DEX implants number = 3 at six months (OR, 1.44; 95% CI, 1.10–1.88; *P* < 0.01) and baseline biomarkers including a CFT ≥ 390 µm (OR, 1.3; 95% CI, 1.05–1.83; *P* = 0.02), total absence or partial disruption of EZ (OR, 1.35; 95% CI, 1.05–1.75; *P* = 0.02), presence of abundant HRD (OR, 1.42; 95% CI, 1.14–1.77; *P* < 0.01), and moderate or severe HE (OR, 1.34; 95% CI, 1.07–1.68; *P* = 0.01). Additionally, severe DME stage was associated with a decrease in CFT ≥ 10% at six months.

**Table 4:** Univariate analysis for predictors of 6-month outcomes in diabetic macular edema treatment with DEX implants^a^.

Variables	Endpoint 1: CFT decreased ≥ 10% from baseline			Endpoint 2: CFT decreased to < 300 µm			Endpoint 3: BCVA improved ≥ 15 ETDRS letters		
	Beta	OR (95% CI)	*p-*value	Beta	OR (95% CI)	*p-*value	Beta	OR (95% CI)	*p-*value
Age	− 0.02	0.99 (0.98–1.01)	0.10	− 0.01	1.00 (0.98–1.01)	0.37	− 0.01	1.00 (0.98–1.02)	0.49
Male	− 0.08	0.94 (0.72–1.23)	0.61	0.07	1.07 (0.83–1.38)	0.61	0.07	1.08 (0.85–1.36)	0.57
HbA1c	− 0.04	0.97 (0.87–1.08)	0.49	0.02	1.02 (0.90–1.15)	0.82	0.06	1.06 (0.97–1.15)	0.25
Prior PRP	− 0.25	0.79 (0.62–1.01)	0.05	− 0.11	0.91 (0.71–1.16)	0.42	− 0.24	0.79 (0.64–0.98)	0.03*
Prior anti-VEGF	0.24	1.27 (0.95–1.71)	0.11	0.25	1.29 (0.90–1.84)	0.17	0.19	1.21 (0.83–1.75)	0.34
DEX implants No. at 6-mo									
DEX implant = 1	Reference			Reference			Reference		
DEX implants = 2	0.22	1.24 (0.93–1.65)	0.14	0.24	1.28 (1.02–1.60)	0.04*	− 0.08	0.93 (0.66–1.30)	0.65
DEX implants = 3	0.37	1.44 (1.10–1.88)	< 0.01**	0.32	1.38 (1.04–1.84)	0.03*	− 0.08	0.92 (0.66–1.30)	0.64
Phakic lens status	0.03	1.03 (0.77–1.37)	0.87	-0.02	0.99 (0.77–1.27)	0.93	0.14	1.15 (0.89–1.48)	0.31
CFT ≥ 390 µm	0.33	1.39 (1.05–1.83)	0.02*	0.06	1.06 (0.83–1.36)	0.66	0.08	1.08 (0.87–1.34)	0.51
Severe IRC (reference: moderate or less IRC)	0.28	1.32 (0.99–1.75)	0.06	0.09	1.09 (0.84–1.41)	0.53	0.02	1.02 (0.78–1.33)	0.91
Disrupted or absent EZ (reference: intact EZ)	0.3	1.35 (1.05–1.75)	0.02*	0.18	1.20 (0.94–1.54)	0.16	0.11	1.11 (0.88–1.4)	0.39
DRIL present	0.19	1.21 (0.88–1.66)	0.26	-0.06	0.95 (0.71–1.27)	0.72	0.15	1.17 (0.95–1.44)	0.17
Abundant HRD	0.35	1.42 (1.14–1.77)	< 0.01**	0.33	1.39 (1.11–1.76)	< 0.01**	0.14	1.15 (1.13–1.49)	0.29
Subretinal fluid present	0.25	1.28 (0.97–1.68)	0.09	0.18	1.20 (0.87–1.66)	0.28	0.06	1.06 (0.78–1.43)	0.74
Detached vitreous cortex in fovea (reference: attached vitreous cortex in fovea)	− 0.3	0.75 (0.49–1.14)	0.17	− 0.29	0.76 (0.54–1.06)	0.10	− 0.37	0.70 (0.50–0.98)	0.03*
Presence of epiretinal membrane	− 0.16	0.86 (0.65–1.10)	0.22	− 0.27	0.77 (0.59–0.99)	0.04*	− 0.13	0.88 (0.68–1.14)	0.34
Moderate or severe HE (reference: absent or mild HE)	0.3	1.34 (1.07–1.68)	0.01*	0.04	1.04 (0.82–1.31)	0.77	− 0.01	1.00 (0.80–1.25)	0.96
DME stage^b^	0.13	1.13 (0.81–1.59)	0.49	− 0.18	0.84 (0.58–1.22)	0.35	0.21	1.23 (0.94–1.61)	0.14
Early DME	Reference			Reference			Reference		
Advanced DME	0.13	1.13 (0.81–1.59)	0.49	− 0.18	0.84 (0.58–1.22)	0.35	0.21	1.23 (0.94–1.61)	0.14
Severe DME	0.45	1.56 (1.13–2.16)	< 0.01**	0.06	1.06 (0.67–1.68)	0.81	0.14	1.15 (0.79–1.69)	0.48

*P < 0.05; **P < 0.01.

^a^Univariate logistic regression analysis.

^b^DME stage was graded according to a previous study^20^.

Abbreviation: *anti-VEGF* anti-vascular endothelial growth factor, *BCVA* best-corrected visual acuity, *CFT* central foveal thickness, *DEX implant* dexamethasone intravitreal implant, *DME* diabetic macular edema, *DRIL* disorganized retinal inner layers, *ETDRS* Early Treatment Diabetic Retinopathy Study, *EZ* ellipsoid zone, *HE* hard exudates, *HRD* Hyperreflective dot, *IRC* intraretinal cyst, *PRP* = panretinal photocoagulation.

For the second anatomical endpoint, CFT reduction to less than 300 µm was significantly associated with two and three DEX implants at six months (OR, 1.28; 95% CI, 1.02–1.60; *P* = 0.04 and OR, 1.38; CI, 1.04–1.84; *P* = 0.03, respectively), and with the biomarker of abundant HRD at baseline (OR, 1.39; 95% CI, 1.16–1.7; *P* < 0.01). An epiretinal membrane at baseline had a negative effect on the CFT < 300 µm endpoint (Table 4).

As for the functional endpoint, two negative predictors were identified, namely eyes with prior PRP and a detached vitreous cortex in the fovea (OR, 0.79; 95% CI, 0.64–0.98; *P* = 0.03 and OR, 0.70; 95% CI, 0.50–0.98; *P* = 0.03, respectively). The eyes with these factors were less likely to improve BCVA to more than three lines at six months after DEX implants.

### Multivariate analysis of the biomarkers related to outcomes after DEX implants

**Figure 1:**
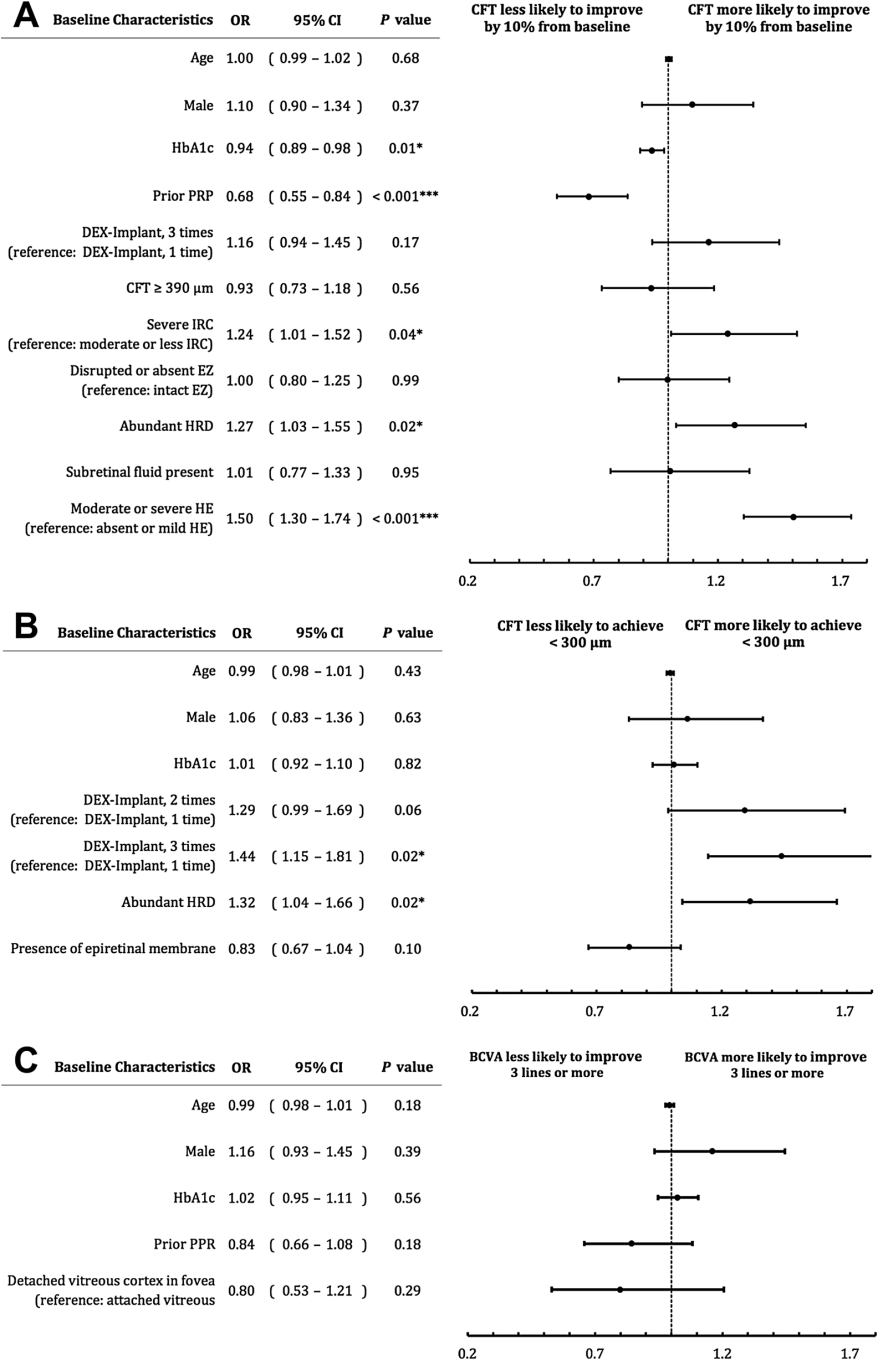
Multivariate analysis of imaging biomarkers and outcomes after dexamethasone (DEX) implant treatment in patients with diabetic macular edema (DME). (**A**) Endpoint 1: CFT reduction of 10% or more from baseline. (**B**) Endpoint 2: CFT < 300 µm at 6-month. (**C**) Endpoint 3: BCVA improvement of 3 lines or more from baseline. (*BCVA* best-corrected visual acuity *CFT* central foveal thickness, *CI* confidence interval, *DEX* dexamethasone, *EZ* ellipsoid zone, *HE* hard exudates, *HRD* Hyperreflective dot, *IRC* intraretinal cyst, *OR* odds ratio, *PRP* panretinal photocoagulation).

Further multivariate analysis of the factors that correlated with six-month outcomes was performed. The results demonstrate that patients with high levels of HbA1c and eyes with prior PRP are less likely to have a CFT reduction ≥ 10% from baseline (OR, 0.94; 95% CI, 0.89–0.98; *P* = 0.01 and OR, 0.68; 95% CI, 0.55–0.84; *P* < 0.001, respectively; Fig. 1A). Biomarkers including severe IRC, abundant HRD, and moderate or severe HE had a significantly higher chance of improving CFT ≥ 10% (OR, 1.24; 95% CI, 1.01–1.52; *P* = 0.04; OR, 1.27; 95% CI, 1.03–1.55; *P* = 0.02 and OR, 1.50; 95% CI, 1.30–1.74; *P* < 0.001, respectively). Furthermore, eyes with abundant HRD at baseline were more likely to achieve the endpoint of CFT < 300 µm (OR, 1.32; 95% CI, 1.04–1.66; *P* = 0.02), as well as eyes that underwent three DEX implants at six months (OR, 1.44; 95% CI, 1.15–1.81; *P* = 0.02; Fig. 1B). Nevertheless, none of the above predictors found in the univariate analysis was significantly correlated with the functional outcome of BCVA improvement ≥ three lines in the multivariate model (Fig. 1C). Although severe DME stage was a significant factor in the univariate analysis, it was not included in the multivariate analysis since it was composed of some other factors included in the model.

## Discussion

In this retrospective, multicenter Asian study, DME patients who were treated with DEX implants had significant anatomical improvement after six months, and their vision improved or stabilized in 79%. Several baseline imaging characteristics including HE, HRD, and IRC were found to be predictive of the significant treatment outcomes. Since OCT is widely available in most clinical settings and is non-invasive, these results provide physicians instant guidance for deciding the individualized treatment options.

For several decades, the assessment of diabetic retinopathy and DME was made by color fundus photography or invasive imaging by fluorescein angiography. The advances in OCT have contributed to understanding the morphological changes, pathophysiology, and classification of DME^11,19,21^. With this technology, the evaluation of various non-invasive imaging biomarkers of DME can be easily performed. Biomarkers such as CFT, choroidal thickness, HRD, HE, SRF, IRC, EZ or external limiting membrane disruption, and DRIL have been shown as general prognostic biomarkers of anatomical or functional outcomes^11,19,21,22^.

Although the pathogenesis of DME is still not completely understood and thought to be multifactorial, there is growing evidence that inflammation plays an important role in the development and worsening of DME^4^. Therefore, biomarkers related to inflammation and DME outcomes are emerging as treatment predictors.

The nature of HRD remained unclear despite several proposed inflammatory origins, including lipid extravasation from a compromised vasculature, microglia proliferation, or retinal pigmented epithelium migration^23^. After anti-VEGF treatment for DME, the results on the relationship between HRD and treatment responses are conflicting. While some studies have reported better VA after anti-VEGF in eyes with more HRD^23^, many others showed no such associations, or even a poorer VA improvement or final VA in eyes with HRD^24^.

Similarly, for the outcomes of treating DME with DEX implants, HRD had different predictive values. Zur et al.^14^ and Chatziralli et al.^15^ reported that the presence of HRD at baseline was related to a worse VA or VA gain after DEX implants. Conversely, in eyes with more HRD, greater improvement in retinal sensitivity was found one month after DEX implant^18^. Additionally, more HRD was found in anti-VEGF non-responders. After switching to DEX implant, a significant reduction in retinal thickness or better VA outcomes were observed in eyes with more HRD^14,16^. A recent report also found that the number of HRD decreased significantly in eyes treated with DEX when compared to those treated with anti-VEGF^25^. Since HRD might be related to inflammation, intravitreal DEX implants may be more effective than anti-VEGF agents in eyes with more HRD^12^. This explains the results from the current study, in which abundant HRD at baseline were associated with a significant reduction in CFT and increased likelihood of achieving a dry macula at six months after DEX implants.

HE develops due to protein and lipid extravasation as a result of increased vascular permeability, vasodilatation, inflammation, and a breakdown of the inner blood-retina barrier; and, if left untreated, might lead to retinal fibrosis^26^. Studies have shown that the presence of HE was associated with improvement of CFT and correlated with VA improvement after anti-VEGF^27^, while other studies reported an absence of association between HE and VA outcomes in anti-VEGF treated eyes^28^. Compared to bevacizumab^29^, DEX implants provided a more rapid regression of HE, and a recent study showed that a decrease in HE area after DEX implants correlated with VA improvement^30^. The current study also showed that eyes with moderate or severe HE have a significantly higher chance of improving CFT > 10%. These effects can be explained by the different mechanisms of action between anti-VEGFs and DEX implants. While anti-VEGF only exhibits anti-vascular permeability effects, DEX is involved in anti-vascular permeability, vasoconstriction, and anti-inflammatory effects^31^.

Lobulated retinal edema including SRF and IRC may also serve as a biomarker for DME. A study showed that the concentrations of interleukin (IL)-6 and IL-8 were significantly higher in eyes with SRF than those without SRF^32^. The inflammatory nature of IRC and the involved cytokines have also been extensively studied and include IL-6, IL-8, monocyte chemoattractant protein (MCP)-1, intercellular adhesion molecule (ICAM)-1, and VEGF among others^33^. An improvement in IRC after DEX implant was reported in previous studies^8,11^, in the current study, we found that severe IRC at baseline was related to a significant reduction in CFT. The association between IRC and inflammatory cytokines may explain why there is a significant response after DEX treatment in the current study, and our results suggest this biomarker as a predictor of anatomical outcomes.

The current study also identified significant negative prognostic biomarkers that predict a worse outcome after DEX treatment. After six months, eyes with prior PRP or patients who had higher baseline HbA1c levels showed worse morphological outcomes, evidenced by a lack of CFT reduction. In line with this, previous studies have shown that patients with prior PRP tended to have poorer final BCVA after DEX implants^34^, and that higher HbA1c levels were correlated with a worse visual outcome in DME patients^15,25^. In our study, 50% of the patients had prior PRP. Nevertheless, DEX implants significantly reduced DME and improved vision in 22% of the patients while significant worsening of vision was noted only in 12% of the patients after six months of DEX implant treatment.

It might be worth noting that no overall mean VA improvement was observed in the current study since this is a real-world study and that half of the patients had prior PRP. In fact, many of the published studies showed a positive functional effect in certain DME subgroups, not in the overall study population. A meta-analysis of 4 randomized trials also demonstrated that DEX implant achieved anatomical improvement but not functional enhancement at 12 months^31^. In recent years, studies utilizing newer imaging modalities including ultra-widefield or OCT angiography showed that DME severity or functional outcomes was related to biomarkers such as retinal vascular bed area or foveal avascular zone of the deep vascular plexus^35,36^. Further studies utilizing those modalities will further elucidate the response of DME to DEX implants. Nevertheless, in eyes affected by DME, morphological improvement paves the road to functional recovery, especially in those poorly responding to anti-VEGF agents.

Finally, this study has several limitations that require consideration. It was a non-randomized retrospective single-arm study with a relatively small number of eyes. The treatments were arranged using a non-unified protocol, and patients received different injections of DEX implants over the period of six months. Also, the higher proportion of patients with previous PRP (> 50%) and the longer mean duration of DME (109 days) indicates that the patient cohort leaned to the more severe and chronic end of diabetic retinopathy, which makes the results of this study less generalizable to the overall DME patients. Still, the strength lies in studying an Asian population from different medical centers. We observed that the number of DEX implants was significantly related to anatomical outcomes; nevertheless, after adjusting the number of DEX implants in the multivariate analyses, we found that other biomarkers are still significantly related to the outcomes.

In conclusion, we identified the predictive value of imaging biomarkers of anatomical outcomes in DME patients following a DEX implant in a real-world, multicenter scenario. Biomarkers including abundant HRD, moderate-to-severe HE, or severe IRC at baseline were more likely to exhibit anatomical improvements after DEX implant, while eyes with prior PRP and underlying higher HbA1C were less likely to improve. In line with this, a recent guideline for DME treatment suggested that eyes with more abundant HRD or HE should consider using DEX implant as the first-line treatment instead of anti-VEGF^37^. Given the anti-inflammatory nature and the ability to target more components of DME pathophysiology, DEX implant should be considered in eyes presenting these inflammatory biomarkers.
